# Sexual Dimorphism in Cellular and Molecular Features in Human ACTH-Secreting Pituitary Adenomas

**DOI:** 10.3390/cancers12030669

**Published:** 2020-03-13

**Authors:** Francesca Pecori Giraldi, Maria Francesca Cassarino, Antonella Sesta, Mariarosa Terreni, Giovanni Lasio, Marco Losa

**Affiliations:** 1 Department of Clinical Sciences & Community Health, University of Milan; 20122 Milan, Italy; 2 Neuroendocrinology Research Laboratory, Istituto Auxologico Italiano, Istituto di Ricerca e Cura a Carattere Scientifico, 20095 Milan, Italy; 3 Deparment of Pathology, Ospedale San Raffaele, 20136 Milan, Italy; mariarosa.terreni@hsr.it; 4 Deparment of Neurosurgery, Istituto Clinico Humanitas, 20089 Rozzano (Milan), Italy; giovanni.lasio@humanitas.it; 5 Department of Neurosurgery, Ospedale San Raffaele, 20136 Milan, Italy; marco.losa@hsr.it

**Keywords:** Cushing’s disease, gender, gene expression profiling, neuroendocrine tumours, ACTH-secreting adenomas

## Abstract

(1) Background. Cushing’s disease presents gender disparities in prevalence and clinical course. Little is known, however, about sexual dimorphism at the level of the corticotrope adenoma itself. The aim of the present study was to evaluate molecular features of ACTH-secreting pituitary adenomas collected from female and male patients with Cushing’s disease. (2) Methods. We analyzed 153 ACTH-secreting adenomas collected from 31 men and 122 women. Adenomas were established in culture and ACTH synthesis and secretion assessed in basal conditions as well as during incubation with CRH or dexamethasone. Concurrently, microarray analysis was performed on formalin-fixed specimens and differences in the expression profiles between specimens from male and female patients identified. (3) Results. ACTH medium concentrations in adenomas obtained from male patients were significantly lower than those observed in adenomas from female patients. This could be observed for baseline as well as modulated secretion. Analysis of corticotrope transcriptomes revealed considerable similarities with few, selected differences in functional annotations. Differentially expressed genes comprised genes with known sexual dimorphism, genes involved in tumour development and genes relevant to pituitary pathophysiology. (4) Conclusions. Our study shows for the first time that human corticotrope adenomas present sexual dimorphism and underlines the need for a gender-dependent analysis of these tumours. Differentially expressed genes may represent the basis for gender-tailored target therapy.

## 1. Introduction

ACTH-secreting pituitary adenomas, i.e., Cushing’s disease, are known to occur far more frequently in women than in men [1,2] and, as we first showed some years ago [3], give rise to a somewhat different clinical course in the two sexes. In fact, men with Cushing’s disease are more likely to be younger, exhibit a more severe clinical presentation and a less favourable response to surgical as well as medical treatment [3,4,5,6,7,8,9]. Furthermore, recently identified somatic mutations in the deubiquitinases *USP8* and *USP48* occurs with greater frequency in women than men with Cushing’s disease [10,11], suggesting that the corticotrope adenoma itself may harbour features which contribute to gender-dependent differences in Cushing’s disease.

Little is known, however, on molecular features of ACTH-secreting adenomas from female and male patients, an avenue of research which may yield novel insights into Cushing’s disease pathophysiology and, possibly, provide the basis for tailored diagnostic and therapeutic approaches. The aim of the present study was to evaluate differences in ACTH-secreting adenomas collected from female and male patients. Our approach to gender differences was two-fold, on the one side we assessed the secretory status of corticotrope adenomas in culture, on the other we compared the gene expression profile in archival adenomatous specimens.

## 2. Results

### 2.1. ACTH Synthesis and Secretion Pattern

Data from 124 adenoma primary cultures was available. Spontaneous ACTH secretion at both 4 h and 24 h was significantly lower in cultures obtained from male compared to female patients (Table 1). Lower ACTH levels were also observed in cultures from male patients during CRH and dexamethasone incubation although the percentage change over baseline was comparable (Table 1). Basal as well as CRH- and dexamethasone-modulated POMC expression was comparable in specimens from female and male patients (Table 1).

### 2.2. USP8 Sequencing

*USP8* sequencing was performed in 111 adenoma specimens and 29 proved carriers of somatic mutations; in detail, 25 adenomas out of 84 specimens tested in culture carried *USP8* variants as did 5 out of 38 specimens used in microarray analysis. All variants have been previously described [10,12]. As expected, the proportion of *USP8* variant carriers was greater in adenomas from female compared to male patients (32.2% vs. 4.2%, *p* < 0.005); in fact, only a single adenoma from a male patient presented an USP8 mutation (i.e., p.P720R); thus, no subanalysis according to *USP8* variant status could be performed. We present a description of the experimental results and their interpretation, as well as the experimental conclusions that can be drawn.

### 2.3. Gene Expression Pattern

To determine the expression pattern common to samples from male or female patients with Cushing’s disease, we first identified gene expressed in samples (9 males and 31 females) from either sex: out of 20,815 genes, 2141 and 1914 were significantly expressed in all female and male specimens, respectively. Analysis of gene lists revealed 1206 genes expressed in both groups, 935 expressed only in specimens from female patients and 708 only in specimens from male patients. Functional annotation in genes expressed in both revealed enrichment in functions related to ribosomal function, protein biosynthesis, vesicle transport groups, similar to previously described annotations in corticotrope adenomas per se [13]. Analysis of the two sets of uniquely expressed genes is shown in Table 2 and Table 3.

Some functions, e.g., mitochondrion, cell–cell adhesion, RNA transcription, were enriched in both gene sets, with different genes associated with the same functional pathway. For example, *JUP* (junction plakoglobin) and *PFN1* (profilin 1) contributed to cell-cell adhesion terms in specimens from male patients and *LIMA1* (LIM domain and acting binding 1) and *ADD1* (adducin 1) contributed to the same term in specimens from female patients. Genes uniquely expressed in female samples could also be annotated to the spliceosome and ribosome KEGG pathways, nucleotide binding, chaperonin and histone de-acetylation GOTERM functions and WD-repeats in Uniprot sequence features. Conversely, in samples from male patients, the iron-sulphur pathway was enriched for Uniprot and GOTERM databases.

**Figure 1:**
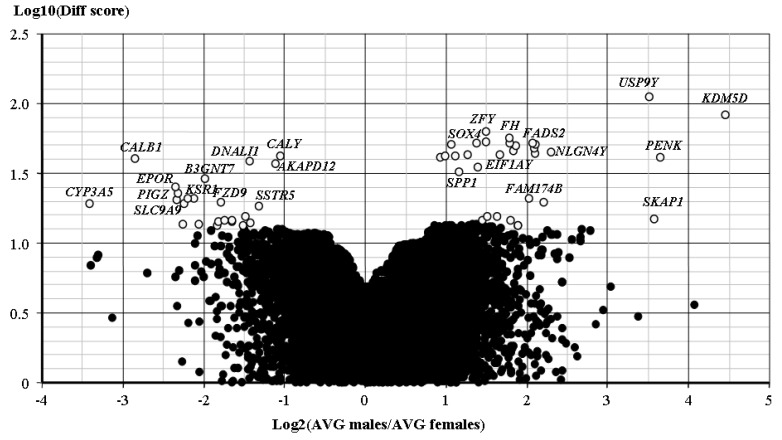
Volcano plot. Genes up- and down-regulated specimens from male vs. female patients. Effect (ratio of average signal, AVG) is shown on the x-axis and significance (Diff Score) on the y-axis. Up-regulated genes appear to the right and down-regulated genes appear to the left on the x-axis. White circles indicate significant genes (Diff Score > 13) and selected genes are identified by name.

Differential expression analysis identified several genes variably expressed according to gender. Genome Studio and Limma algorithms yielded comparable results: overall, 31 genes were overexpressed in samples from male patients and 24 genes in samples from female patients (Table 4, Figure 1).

Several genes encoded on chromosome Y were overexpressed in samples from male patients, e.g., *USP9Y*, *KDM5D*, *EIF1AY*, *ZFY*, *TTTY14*, *NLGN4Y*. Conversely, none of the genes overexpressed in adenomas collected from female patients are encoded on the X chromosome. Analysis of differently expressed genes revealed that several genes detected at higher levels in specimens from male or female patients are known to present tissue-dependent sexual dimorphism, e.g., *CALB1*, *SPP1*, *PENK*. Among genes overexpressed in samples from male patients a considerable number are associated with tumourigenesis, e.g, *FH*, *NETO2*, *NXP2*, *PDLIM2*, *PTMA*, whereas other genes, such as *SOX4* and *SPP1*, are involved in pituitary pathophysiology. *SSTR5*, encoding for the somatostatin receptor subtype 5, was overexpressed in samples from female patients (Table 4). Conversely, the other somatostatin receptor subtypes were not differentially expressed and mean expression in samples from male and female patients was comparable (ratio average normalized expression 1.01, 1.02, 0.93 and 0.97 for *SSTR1*, *SSTR2*, *SSTR3* and *SSTR4*, respectively, all diff-scores N.S.). None of the major oestrogen-responsive genes [14,15] proved to be overexpressed in samples from female patients.

## 3. Discussion

Since its first description by Harvey Cushing [1], ACTH-secreting adenomas rank among the few tumours with female preponderance. Indeed, with the notable exception of neoplasias in reproductive organs only few tumours, e.g., meningiomas, thyroid carcinomas, occur more frequently in women than in men [16,17]. The issue of gender differences in tumour susceptibility has been the focus of an increasing number of studies, revealing differences in immune surveillance, mutations, epigenetic patterns and, ultimately, gene expression [18,19,20].

One major contributor to sexual dimorphism is sex hormone-related pathways; in fact, the role of oestrogen in female reproductive organs [21,22] and testosterone in prostate cancer [21,23] has been clearly established, to the point that it represents the basis for target therapy. Oestrogen also has been implicated in melanoma [24] and papillary thyroid cancer development [25] whereas progesterone appears to play a role in meningiomas [26], leading to trials with mifepristone, the progesterone receptor antagonist [27].

As regards the pituitary, prolactin-secreting adenomas can be induced in animals by prolonged oestrogen treatment [28,29] and prevented by oestrogen receptor agonists [30]. In vitro, oestradiol stimulated both lactotrope, somatotrope and corticotrope proliferation [31,32], while testosterone appeared to inhibit proliferation in lactotrope and gonadotrope adenomas [31]. Oestrogen receptors have also been linked to aggressiveness in non-functioning pituitary adenomas [33] and in pancreatic neuroendocrine tumours [34]. Several studies sought oestrogen receptors by immunohistochemistry in corticotrope adenomas, but expression appears far less than in other pituitary adenomas [35,36,37]. Conversely, immunohistochemical analysis revealed that over 50% of corticotrope adenomas and normal corticotrope cells express the androgen receptor [38].

From a clinical viewpoint, in addition to clear preference for the female sex [2], clinical presentation and course differ between men and women with Cushing’s disease. In fact, our first report on gender-dependent differences among these patients [3] was subsequently confirmed by other investigators [6,7,8,9]. Male patients with Cushing’s disease usually present at a younger age, with more severe hypercortisolism and pronounced clinical features; hypogonadism induced by cortisol excess appears an important contributor to some clinical signs in males [8,39,40]. As regards hormonal secretion, urinary free cortisol levels are higher in male patients with Cushing’s disease [3,6] as occurs in normal adult men [41,42]; plasma ACTH concentrations follow the same pattern as higher levels have been observed in male patients [3,6,7,9], as well as in normal men [42,43]. Comparison of responses to diagnostic tests revealed that men with Cushing’s disease are less likely to inhibit with the high dose dexamethasone test [3,7,8] and less likely to present positive pituitary magnetic resonance imaging [3,6]; in fact, inferior petrosal sinus sampling was required more frequently in men than women to confirm the pituitary lesion [44]. Of note, a higher prevalence of pituitary macroadenomas in male patients has been reported in two Chinese series [7,8], thus there might by ethnic diversity in sexual dimorphism of corticotrope adenoma size. In addition to the more complex diagnostic work-up, men with Cushing’s disease present less favourable surgical outcomes and higher risk of recurrence after successful surgery [3,4,8,45]. Control of hypercortisolism by kecotonazole, one of the mainstays of medical therapy in Cushing’s disease, also proved worse in male compared to female patients [5]. These abovementioned findings were mostly reported in adults with Cushing’s disease, as the difference in both prevalence and clinical features was less pronounced in children with pituitary ACTH-secreting adenomas [46,47], again underlying the potential contribution of sex hormones to presentation of Cushing’s disease.

Interestingly, exome sequencing recently identified two somatic mutations, i.e., *USP8* and *USP48*, which occurred with far greater frequency in corticotrope adenomas from women with Cushing’s disease [10,11], thus indicating that the corticotrope adenoma itself may harbour features which contribute to gender-dependent differences in Cushing’s disease. In fact, we and others observed different molecular signatures in adenomas carrying *USP8* variants compared to *USP8*-wildtype adenomas [12,48,49], and this carried over into increased POMC synthesis and ACTH secretion [12] and changes in intracellular signalling [50].

Our study aimed to identify differences in cellular and molecular features in adenomas collected from male and female patients with Cushing’s disease and can indeed report on several, relevant differences. These results are of major interest given that most studies on corticotrope adenomas were performed on specimens from women with Cushing’s disease—quite inevitable, given the skewed gender prevalence—and thus the findings most likely reflect features of female corticotrope adenomas rather than corticotrope adenomas per se. Of note, only corticotrope adenomas from patients with features of hypercortisolism were included in the study.

One major finding relates to ACTH secretion as specimens from male patients secreted considerably less ACTH than their female counterparts. Spontaneous ACTH secretion in adenomas from male patients was less than half the concentrations measured in adenomas from women at 4 h and nearly one third at 24 h. Furthermore, corticotrope adenomas from males secreted less ACTH in response to CRH stimulation compared to females, although the percent change from baseline was comparable between sexes. In addition, medium ACTH concentrations during incubation with dexamethasone were tenfold less in corticotrope cultures from male patients compared to female patients. Again, the percent change from baseline during dexamethasone did not differ between sexes, indicating proportionality in the response to dexamethasone. These in vitro results are inverse to in vivo findings, in fact, as mentioned above, ACTH plasma levels are usually higher in men than in women with Cushing’s disease, and men present a lesser response to dexamethasone inhibition. On the other hand, plasma ACTH is an unreliable marker of corticotrope tumour activity [42] and, indeed, cortisol rather than ACTH represents the parameter for diagnosis and treatment monitoring in Cushing’s disease [51,52]. It follows that only results obtained in corticotrope tumour primary cultures reveal the secretory features of these adenomas.

As regards ACTH synthesis, we did not observe differences in *POMC* expression between male- and female-excised adenomas both in unchallenged wells and after CRH/dexamethasone incubation. This finding is in line with the lack of correlation between *POMC* and ACTH in corticotrope adenomas in vitro [53,54] and, in the present setting, suggests that sexual dimorphism affects POMC peptide processing and secretion rather than *POMC* transcription.

Analysis of gene expression in specimens from female and male patients with Cushing’s disease revealed some uniquely expressed genes in the context of considerable similarities between the sexes. In fact, evaluation of significantly expressed genes showed that over 50% of genes were expressed in both female- and male-derived adenomas and, further, that several uniquely expressed genes concurred to the same cellular function. For example, both groups were enriched for cell–cell adhesion, mitochondrion and RNA processing although annotated genes differed. These differences, as well as gender-distinctive annotations, provide clues as to sexual dimorphism in tumoural susceptibility. In this context, studies on the role of oestrogen on breast cells illustrated the relationship between the oestrogen-regulated transcriptome and the mitogenic response [55]. Conversely, the androgen receptor mediates the angiogenetic and immune response to neoplasia in several tumour models [17,56]. The role of sex hormones on these differences in functional annotations remains to be established.

Differential gene expression analysis proved significant for a small number of genes, 31 and 24 in samples from male and female patients, respectively, approximately 0.2% out of the entire gene expression set. This percentage is in line with results obtained in normal tissues and blood cells [57,58,59] and in some cancers, e.g., colon adenocarcinoma, acute myeloid leukemia [57,60]; conversely, up to 14% of genes were differentially expressed according to sex in other tumours, such as kidney clear cell carcinoma, thyroid carcinoma, liver hepatocellular carcinoma [57,60], suggesting marked diversity in sexual dimorphism across neoplasias.

Nearly 30% of genes overexpressed in adenomas from male patients are encoded in the Y chromosome whereas none of the genes overexpressed in female originate from the X chromosome (see Table 4). All protein coding genes are X-Y homologues and reside in the AZF locuses [61]. Given that only approximately 4% of genes originate from sex chromosomes, adenomas from male patients are enriched in genes from the Y chromosome. Several of these genes, e.g., *EIF1AY*, *USP9Y*, *ZFY*, *TMSB4Y*, have been used as gender-specific tissue biomarkers for both normal and tumoural tissues [57,58,62,63]; corticotrope adenomas can now be added to the list of tissues presenting these markers of sexual dimorphism.

In addition to sex chromosome-encoded genes, several autosomal genes were up-regulated in adenomas from male patients; of note, *PDLIM2*, *PENK*, *SOX4*, *FH*, *PTMA* and *TMEM97* are all associated with tumourigenesis [64,65,66,67,68,69], and thus could play a role in the less favourable course of corticotrope adenomas in male patients. Links between these factors and the pituitary are known for *PTMA* and *SOX4*, as *PTMA* nuclear staining has been linked to pituitary tumour size [70], and *SOX4* is involved in pituitary development, as shown in both zebrafish [71] and human tissues [72]. Along the same line, another gene overexpressed in male-derived adenomas associated with the pituitary is *SPP1* (osteopontin) with increased expression reported in ACTH-secreting adenomas compared to non tumourous pituitary tissue [73] and in corticotrope and lactotrope adenomas compared to other pituitary tumours [49]. Interestingly, *SPP1* is known to present sexual dimorphism as greater expression was observed in male compared to female rat pituitaries [74] and oestrogens have been shown to modulate *SPP1* expression in a variety of tissues [75,76]. Another gene of interest is *PENK*, i.e., proenkephalin, part of the POMC-derived opioid family [77]. In addition to the abovementioned role in tumourigenesis [68], there is evidence for its modulation by glucocorticoids [78], gender-distinct expression [79] and involvement in the hypothalamo-pituitary-adrenal axis [80].

On the other hand, among genes overexpressed in adenomas from female patients, *AKAP12* (gravin) is a known tumour suppressor [81] and *SLC9A9* is associated with epidermal growth factor (EGF) receptor turnover [82], EGF itself notably involved in corticotrope tumourigenesis [83,84]. Overexpression of *FZD9*, a receptor to Wnt proteins, further links adenomas from female patients with EGF, as the EGFR pathway interacts with Wnt/ßcatenin signalling [85]. Another gene overexpressed in samples from female patients is *EPOR*, i.e., the erythropoietin receptor; given that erythropoietin has been shown to modulate ACTH intracellular concentration and secretion in AtT 20 cells [86]; this finding could contribute to the gender-dependent difference ACTH secretion by corticotrope adenoma primary cultures. Interestingly, somatostatin receptor subtype 5 was also among genes expressed with greater abundance in adenomas from female patients. Assessment of somatostatin receptor in corticotrope adenomas had revealed that *SSTR5* is the most abundant receptor isoform [87,88]. Interest in *SSTR5* rests on the fact that that pasireotide, a somatostatin analogue with affinity for several somatostatin receptor subtypes including SSTR5, is being used to contain tumoural corticotrope secretion. Clinical efficacy of subcutaneous pasireotide is possibly superior in women [89], but gender-skewed sample collection—the vast majority of samples were from female donors—might have influenced this result. Overexpression of *SSTR5* has recently been reported among *USP8*-mutated corticotrope adenomas compared to wild-type adenomas [49]; however, *USP8* mutations were found in adenomas from female patients only, thus this finding could be gender- rather than *USP8*-variant specific. In fact, findings reported so far on *USP8* variant adenomas [12,48,49,50] were collected almost exclusively in female patients; only two specimens were obtained from male patients, the remainder (80 *USP8* variant adenomas in the four series) in female patients. A multicentre effort is clearly required to discern sex-independent, *USP8*-determined features.

Furthermore, among genes overexpressed in female samples is calbindin (*CALB1*), a calcium-binding protein expressed within the brain with known sexual dimorphism [90]. In fact, oestrogen and androgen treatment or receptor blockade are known to affect calbindin expression in the preoptic area, cortex and cerebellum [90]. Calbindin is expressed in the mouse developing pituitary and appears to localize mainly in corticotropes and somatotropes [91]; in adult rats, calbindin staining was stronger in somatotrope cells from male animals and corticotrope and lactotrope cells from female animals [92], suggesting gender-dependent differences in calcium signalling. Further to genes overexpressed in specimens from female patients is *CYP3A5*, encoding for one of the major drug-metabolizing cytochromes [93]. *CYP3A5* is induced by corticosteroids in the liver and can both induce drug resistance and activate prodrugs; it is the subject of ongoing studies in several neoplasias [93] and could represent a viable drug target for corticotrope adenomas from female patients. Interestingly, *CYP3A5* together with *CALB1*, *DAPL1* and *FZD9* were recently reported to be enriched in human ACTH-secreting adenomas compared to other pituitary adenomas [49]. Given our findings, these results are likely to reflect predominance of female specimens in the series (22 vs. 5 from men) in lieu of lineage-specific features.

## 4. Materials and Methods

### 4.1. Specimens

One hundred fifty-three ACTH-secreting pituitary adenomas were collected during transsphenoidal surgery for Cushing’s disease. All tumours fulfilled criteria for “corticotrope adenoma” (8272/0) according to WHO 2017 Classification of Pituitary Tumours [94]; null cell and silent corticotrope adenomas were not included. The diagnosis of Cushing’s disease had been established by standard criteria [51,95]. Our specimen collection comprised 31 men and 122 women, aged from 14 to 76 years (median 40.1 years). No significant differences as regards surgical outcome and adenoma size were detected between sexes; men were slightly older than women (see Table 5). Presurgical medical treatment was reported in 12 (4 men) out of 117 patients in whom this information could be established; six patients had been treated with ketoconazole, five with cabergoline and one patient with s.c. pasireotide; all drugs had been interrupted at least 3 days prior to surgery. MIB-1 staining was <2% in all but one specimen; this adenoma presented MIB-1 index 9%, mitosis count 3 per 10 high power field and had been collected from a female patient in whom surgery proved successful. As per our previous publications [53,96,97], the presence of corticotrope cells in fresh adenoma specimens was assured by abundant ACTH secretion in culture medium [98]; as regards formalin-fixed specimens, abundant *POMC* and absent *GH*, *PRL*, *PIT1*, *LHB*, *FSHB* expression was documented by microarray analysis [13].

### 4.2. Human Pituitary Adenoma Primary Culture

Specimens were established in culture according to our standard protocol [98,99]; primary cultures were incubated in serum-free DMEM + 0.1% bovine serum albumin (BSA) containing 10 nM CRH or 10 nM dexamethasone. Wells treated with DMEM + BSA only represented control secretion. Medium was collected after 4 h and 24 h for measurement of ACTH; after 24 h, RNA was extracted using Pure Link RNA mini kit (Invitrogen, Carlsbad, CA, USA).

### 4.3. ACTH Assay

ACTH was measured by immunometric assay (Diasorin S.p.A. Saluggia, Italy) with all samples from a given specimen assayed in the same run. Intra-assay coefficient of variation is 7.9% and assay sensitivity 1.2 pg/mL. Given the considerable variability in ACTH adenoma concentrations [98], ACTH concentrations were normalized to number of cells per well and responses to CRH and dexamethasone expressed relative percent of secretion in wells incubated with DMEM + BSA only (control = 100%) for statistical analyses. Plated cells counts were comparable between specimens from female and male patients (138,305 ± 18,460 vs. 110,449 ± 23,372 cells per well, respectively, N.S.)

### 4.4. Microarray Analysis from Archival Specimens

RNA was extracted from formalin-fixed paraffin-embedded adenomatous samples using Recover All Total Nucleic Acid Isolation Kit (Life Technologies, Carlsbad, CA, USA), as previously described [12,13]. RNA (300 ng) was analysed on Human HT_12 v4 Bead Chip (Whole Genome DASL High Throughput assay, Illumina, San Diego, CA, USA) and fluorescence data captured into HiScan, a high-resolution laser imager (Illumina). Array data has been deposited at https://www.ncbi.nlm.nih.gov/geo/query/acc.cgi?token=itsvwwkuzjyvpsj&acc=GSE93825.

### 4.5. Differential Gene Expression Analysis

Two approaches were used to identify differences in gene expression patterns. First, we identified the expression pattern common to adenomas from either sex. Genome studio software (Illumina) was used to identify genes significantly expressed, i.e., detection *p* value < 0.01, in all specimens from either male or female patients and the two lists were compared for genes expressed in both or either group. Second, differential expression across all probes was analysed by Genome Studio and Limma [100]. Expression was analysed after quantile normalization and genes with Benjamini-Hochberg *p* < 0.05 were considered significant. Diff Scores were calculated based on p value transformation according to the difference between average signals in specimens from male and female patients with Cushing’s disease. Volcano plot [101] was used to illustrate differential expression.

### 4.6. Functional Annotation and Gene Ontology

DAVID v6.7 [102] was used to annotate and classify significant genes and perform functional annotation clustering. Minimum value of enrichment score for significant clusters was 1.3. Clusters were annotated to Gene Ontology (GO) project, Kyoto Encyclopedia of Genes and Genomes (KEGG) and Protein Information Resource (SP_PIR).

### 4.7. USP8 Sequencing

RNA was obtained from formalin-fixed or fresh specimens and carried out as previously described [12,103].

### 4.8. Real-Time PCR

RNA (100 ng) was reverse transcribed (Superscript-Vilo cDNA synthesis kit; Life Technologies) and quantitative Real-Time PCR performed on 7900 HT sequence Detection System (Applied Biosystem, Foster City, CA, USA), using Platinum Quantitative PCR Supermix-UDG with premixed ROX. Taqman assay (Applied Biosystem) was used for *POMC* quantification (probe Hs00174947_m1). Basal expression data (2^−ΔCt^) was calculated and normalized to *RPLP0* (probe Hs99999902_m1); expression after treatments was analysed as 2^−ΔΔCt^ and expressed in fold change from baseline [53].

### 4.9. Ethics

The study was conducted in accordance with the Declaration of Helsinski and the protocol approved by the Ethical Committee of the Istituto Auxologico Italiano (protocol 02C102_2011 approved on 12/04/2011 and protocol 02C402_2014 approved on 4/3/2014). Informed consent for the use of secondary surgical materials was granted by patients prior to surgery.

### 4.10. Statistical Analysis

Differences between specimens from female and male patients with Cushing’s disease were established by ANOVA, Mann-Whitney test, chi-square test or Fisher’s exact test, as appropriate, using Statview 4.5 (Abacus Concepts, Berkeley CA, USA). Significance was accepted for *p* < 0.05 and data is given as mean ± S.E.M.

## 5. Conclusions

In conclusion, our study is the first to report on sexual dimorphism in molecular and cellular features in human ACTH-secreting pituitary adenomas and provides the basis for novel, gender-dependent perspective on the pathophysiology of Cushing’s disease. Future studies on corticotrope tumours have to take sexual dimorphism into account and, possibly, identify gender-tailored therapeutic approaches.

## Figures and Tables

**Table 1:** Secretory pattern in adenomas from female and male patients with Cushing’s disease.

Parameter	Female Patients	Male Patients
ACTH 4 h baseline (ng/100,000 cells)	8.26 ± 1.76	3.26 ± 1.69 *
ACTH 4 h DEX (ng/100,000 cells)	7.18 ± 2.03	0.87 ± 0.39 *
ACTH 4 h CRH (ng/100,000 cells)	30.98 ± 8.37	12.79 ± 6.63 *
ACTH 24 h baseline (ng/100,000 cells)	25.35 ± 5.88	7.94 ± 3.61 *
ACTH 24 h DEX (ng/100,000 cells)	16.69 ± 5.02	1.49 ± 0.44 *
ACTH 24 h CRH (ng/100,000 cells)	63.49 ± 14.61	50.42 ± 29.76
ACTH 4 h % change with CRH	395.09 ± 71.11	323.59 ± 98.76
ACTH 24 h % change with DEX	116.18 ± 24.18	114.76 ± 18.59
*POMC* baseline (relative to *RPLP0*)	98.2 ± 29.41	74.69 ± 62.93
*POMC* 24 h CRH (ratio vs. baseline)	1.65 ± 0.16	3.44 ± 1.38
*POMC* 24 h DEX (ratio vs. baseline)	0.83 ± 0.08	0.67 ± 0.27

* *p* < 0.05 vs. specimens from female patients.

**Table 2:** Functional annotation of genes expressed uniquely in specimens from male patients with Cushing’s disease.

Category	Term	Count	*p* Value
**Annotation Cluster 1**	**Enrichment Score: 2.5871443516464256**		
UP_KEYWORDS	Transit peptide	32	0.0001
UP_SEQ_FEATURE	transit peptide:Mitochondrion	30	0.0002
GOTERM_CC_DIRECT	GO:0005743~mitochondrial inner membrane	26	0.0021
GOTERM_CC_DIRECT	GO:0005759~mitochondrial matrix	20	0.0054
UP_KEYWORDS	Mitochondrion	45	0.0165
GOTERM_CC_DIRECT	GO:0005739~mitochondrion	51	0.0670
**Annotation Cluster 2**	**Enrichment Score: 1.5413963866565796**		
UP_KEYWORDS	Iron-sulfur	7	0.0067
UP_KEYWORDS	4Fe-4S	5	0.0196
GOTERM_MF_DIRECT	GO:0051539~4 iron, 4 sulfur cluster binding	5	0.0406
UP_KEYWORDS	Iron	14	0.1282
**Annotation Cluster 3**	**Enrichment Score: 1.535426320231244**		
UP_KEYWORDS	Iron-sulfur	7	0.0067
GOTERM_MF_DIRECT	GO:0051537~2 iron, 2 sulfur cluster binding	4	0.0412
UP_KEYWORDS	2Fe-2S	3	0.0899
**Annotation Cluster 4**	**Enrichment Score: 1.384474121480598**		
GOTERM_CC_DIRECT	GO:0005913~cell-cell adherens junction	18	0.0202
GOTERM_MF_DIRECT	GO:0098641~cadherin binding involved in cell-cell adhesion	16	0.0374
GOTERM_BP_DIRECT	GO:0098609~cell-cell adhesion	14	0.0931
**Annotation Cluster 5**	**Enrichment Score: 1.340383397277057**		
GOTERM_BP_DIRECT	GO:0006362~transcription elongation from RNA polymerase I promoter	5	0.0144
GOTERM_BP_DIRECT	GO:0045815~positive regulation of gene expression, epigenetic	6	0.0469
GOTERM_BP_DIRECT	GO:0006363~termination of RNA polymerase I transcription	4	0.0745
GOTERM_BP_DIRECT	GO:0006361~transcription initiation from RNA polymerase I promoter	4	0.0864

**Table 3:** Functional annotation of genes expressed uniquely in specimens from female patients with Cushing’s disease.

Category	Term	Count	*p* Value
**Annotation Cluster 1**	**Enrichment Score: 4.211755780679299**		
GOTERM_MF_DIRECT	GO:0098641~cadherin binding involved in cell-cell adhesion	29	0.0000
GOTERM_BP_DIRECT	GO:0098609~cell-cell adhesion	27	0.0001
GOTERM_CC_DIRECT	GO:0005913~cell-cell adherens junction	29	0.0001
**Annotation Cluster 2**	**Enrichment Score: 3.9643562287208445**		
GOTERM_BP_DIRECT	GO:0000398~mRNA splicing, via spliceosome	28	0.0000
UP_KEYWORDS	mRNA splicing	27	0.0000
UP_KEYWORDS	Spliceosome	17	0.0000
UP_KEYWORDS	mRNA processing	30	0.0000
GOTERM_CC_DIRECT	GO:0071013~catalytic step 2 spliceosome	14	0.0001
GOTERM_BP_DIRECT	GO:0008380~RNA splicing	18	0.0006
GOTERM_CC_DIRECT	GO:0005681~spliceosomal complex	11	0.0045
KEGG_PATHWAY	hsa03040:Spliceosome	11	0.0847
**Annotation Cluster 3**	**Enrichment Score: 3.2395038273067827**		
UP_KEYWORDS	Mitochondrion	77	0.0000
UP_SEQ_FEATURE	transit peptide: Mitochondrion	34	0.0011
UP_KEYWORDS	Transit peptide	34	0.0034
GOTERM_CC_DIRECT	GO:0005759~mitochondrial matrix	19	0.1097
**Annotation Cluster 4**	**Enrichment Score: 2.823640223068603**		
UP_KEYWORDS	Ribonucleoprotein	30	0.0000
UP_KEYWORDS	Ribosomal protein	20	0.0001
GOTERM_BP_DIRECT	GO:0006412~translation	24	0.0004
GOTERM_MF_DIRECT	GO:0003735~structural constituent of ribosome	22	0.0004
GOTERM_CC_DIRECT	GO:0005840~ribosome	18	0.0004
GOTERM_BP_DIRECT	GO:0000184~nuclear-transcribed mRNA catabolic process, nonsense-mediated decay	15	0.0004
KEGG_PATHWAY	hsa03010:Ribosome	16	0.0014
GOTERM_BP_DIRECT	GO:0006614~SRP-dependent cotranslational protein targeting to membrane	12	0.0018
GOTERM_BP_DIRECT	GO:0019083~viral transcription	12	0.0068
GOTERM_BP_DIRECT	GO:0006364~rRNA processing	18	0.0080
GOTERM_BP_DIRECT	GO:0006413~translational initiation	12	0.0275
GOTERM_CC_DIRECT	GO:0022625~cytosolic large ribosomal subunit	7	0.0557
GOTERM_CC_DIRECT	GO:0022627~cytosolic small ribosomal subunit	4	0.3048
**Annotation Cluster 5**	**Enrichment Score: 2.56685352683656**		
UP_KEYWORDS	Nucleotide-binding	96	0.0003
UP_KEYWORDS	ATP-binding	77	0.0005
UP_SEQ_FEATURE	nucleotide phosphate-binding region:ATP	55	0.0064
GOTERM_MF_DIRECT	GO:0005524~ATP binding	82	0.0072
UP_KEYWORDS	Kinase	39	0.0257
**Annotation Cluster 6**	**Enrichment Score: 1.9383888797605597**		
INTERPRO	IPR000089:Biotin/lipoyl attachment	4	0.0062
INTERPRO	IPR011053:Single hybrid motif	4	0.0082
UP_KEYWORDS	Lipoyl	3	0.0128
INTERPRO	IPR003016:2-oxo acid dehydrogenase, lipoyl-binding site	3	0.0147
GOTERM_BP_DIRECT	GO:0046487~glyoxylate metabolic process	5	0.0213
**Annotation Cluster 7**	**Enrichment Score: 1.818526737361256**		
GOTERM_MF_DIRECT	GO:0019888~protein phosphatase regulator activity	7	0.0010
GOTERM_MF_DIRECT	GO:0008601~protein phosphatase type 2A regulator activity	6	0.0014
GOTERM_MF_DIRECT	GO:0051721~protein phosphatase 2A binding	5	0.0240
GOTERM_CC_DIRECT	GO:0000159~protein phosphatase type 2A complex	4	0.0445
GOTERM_BP_DIRECT	GO:0050790~regulation of catalytic activity	4	0.5201
**Annotation Cluster 8**	**Enrichment Score: 1.8075356513460852**		
KEGG_PATHWAY	hsa04728:Dopaminergic synapse	16	0.0007
KEGG_PATHWAY	hsa04261:Adrenergic signaling in cardiomyocytes	12	0.0521
KEGG_PATHWAY	hsa04071:Sphingolipid signaling pathway	10	0.0998
**Annotation Cluster 9**	**Enrichment Score: 1.4944394402931314**		
INTERPRO	IPR025995:RNA binding activity-knot of a chromodomain	3	0.0215
INTERPRO	IPR016197:Chromo domain-like	5	0.0374
GOTERM_BP_DIRECT	GO:0016575~histone deacetylation	6	0.0410
**Annotation Cluster 10**	**Enrichment Score: 1.3698761413023455**		
GOTERM_MF_DIRECT	GO:0044183~protein binding involved in protein folding	4	0.0183
INTERPRO	IPR027413:GroEL-like equatorial domain	4	0.0201
GOTERM_BP_DIRECT	GO:1904874~positive regulation of telomerase RNA localization to Cajal body	4	0.0223
INTERPRO	IPR027409:GroEL-like apical domain	4	0.0241
INTERPRO	IPR002423:Chaperonin Cpn60/TCP-1	4	0.0241
GOTERM_BP_DIRECT	GO:1904871~positive regulation of protein localization to Cajal body	3	0.0408
INTERPRO	IPR002194:Chaperonin TCP-1, conserved site	3	0.0476
GOTERM_CC_DIRECT	GO:0005832~chaperonin-containing T-complex	3	0.0483
INTERPRO	IPR027410:TCP-1-like chaperonin intermediate domain	3	0.0806
INTERPRO	IPR017998:Chaperone tailless complex polypeptide 1 (TCP-1)	3	0.0806
GOTERM_MF_DIRECT	GO:0051082~unfolded protein binding	9	0.0861
GOTERM_BP_DIRECT	GO:0032212~positive regulation of telomere maintenance via telomerase	4	0.1459
**Annotation Cluster 11**	**Enrichment Score: 1.3267539609067143**		
GOTERM_BP_DIRECT	GO:0042752~regulation of circadian rhythm	7	0.0153
UP_KEYWORDS	Biological rhythms	11	0.0184
GOTERM_BP_DIRECT	GO:0032922~circadian regulation of gene expression	6	0.0870
GOTERM_BP_DIRECT	GO:0043153~entrainment of circadian clock by photoperiod	3	0.2007
**Annotation Cluster 12**	**Enrichment Score: 1.3244168484975751**		
UP_KEYWORDS	Glycogen metabolism	5	0.0148
UP_KEYWORDS	Carbohydrate metabolism	8	0.0611
GOTERM_BP_DIRECT	GO:0005977~glycogen metabolic process	4	0.1174
**Annotation Cluster 13**	**Enrichment Score: 1.322070667802913**		
UP_SEQ_FEATURE	domain:Leucine-zipper	11	0.0118
SMART	SM00338:BRLZ	6	0.0390
INTERPRO	IPR004827:Basic-leucine zipper domain	6	0.0636
UP_SEQ_FEATURE	DNA-binding region:Basic motif	10	0.1765
**Annotation Cluster 14**	**Enrichment Score: 1.302497203152933**		
UP_SEQ_FEATURE	repeat:WD 5	17	0.0212
UP_SEQ_FEATURE	repeat:WD 7	12	0.0315
INTERPRO	IPR020472:G-protein beta WD-40 repeat	9	0.0324
UP_SEQ_FEATURE	repeat:WD 4	17	0.0380
SMART	SM00320:WD40	17	0.0384
INTERPRO	IPR017986:WD40-repeat-containing domain	20	0.0388
UP_SEQ_FEATURE	repeat:WD 6	14	0.0398
UP_SEQ_FEATURE	repeat:WD 3	17	0.0566
UP_KEYWORDS	WD repeat	17	0.0616
UP_SEQ_FEATURE	repeat:WD 1	17	0.0714
UP_SEQ_FEATURE	repeat:WD 2	17	0.0714
INTERPRO	IPR001680:WD40 repeat	17	0.0717
INTERPRO	IPR015943:WD40/YVTN repeat-like-containing domain	20	0.0725
INTERPRO	IPR019775:WD40 repeat, conserved site	11	0.1285

**Table 4:** Genes differentially regulated according to sex.

*Genes Up-Regulated in Adenomas from Male Patients*			
**SYMBOL**	**DiffScore**	**Chr**	**DEFINITION**
*B3GALT1*	45,498	2	beta 1,3-galactosyltransferase 1
*C3*	40,959	19	complement component 3
*EIF1AY*	21,706	Y	eukaryotic translation initiation factor 1A, Y-linked
*FADS2*	52,155	11	fatty acid desaturase 2
*FAM174B*	19,587	15	family with sequence similarity 174, member B
*FGF5*	47,607	4	fibroblast growth factor 5
*FH*	15,527	1	fumarate hydratase
*HAPLN3*	42,829	15	hyaluronan and proteoglycan link protein 3
*ISG20*	13,330	15	interferon stimulated exonuclease gene 20 kDa
*JAM2*	41,419	21	junctional adhesion molecule 2
*KDM5D*	83,236	Y	lysine demethylase 5D (former *JARID1D*)
*MAP4K2*	14,458	11	mitogen-activated protein kinase kinase kinase kinase 2
*MIR612*	49,436		microRNA 612
*NETO2*	42,006	16	neuropilin (NRP) and tolloid (TLL)-like 2
*NLGN4Y*	43,635	Y	neuroligin 4, Y-linked
*NXPH2*	51,966	2	neurexophilin 2
*PDLIM2*	41,894	8	PDZ and LIM domain 2 (mystique)
*PENK*	40,750	8	proenkephalin
*PTMA*	13,638	2	prothymosin alpha
*SKAP1*	14,899	17	src kinase associated phosphoprotein 1
*SOX4*	51,205	6	SRY (sex determining region Y)-box 4
*SPP1*	43,227	4	secreted phosphoprotein 1 (osteopontin)
*THAP12*	14,458	11	THAP domain containing 12 (former *PRKRIR*)
*TMEM97*	15,527	17	transmembrane protein 97 (former *MAC30*)
*TMSB4Y*	50,840	Y	thymosin beta 4, Y-linked
*TTTY14*	51,522	Y	testis-specific transcript, Y-linked 14
*TXLNGY*	44,773	Y	taxilin gamma pseudogene, Y-linked
*USP9Y*	38,904	Y	ubiquitin specific peptidase 9, Y-linked
*WFS1*	20,982	4	Wolfram syndrome 1 (wolframin)
*ZFY*	62,438	Y	zinc finger protein, Y-linked
*ZNF256*	13,638	19	zinc finger protein 256
*Genes up-regulated in adenomas from female patients*			
**SYMBOL**	**DiffScore**	**Chr**	**DEFINITION**
*AKAP12*	−35,303	6	A kinase anchor protein 12 (gravin)
*ANKRD24*	−13,814	19	ankyrin repeat domain 24
*ATAD2*	−20,693	8	ATPase family, AAA domain containing 2
*B3GNT7*	−13,766	2	eta-1,3-N-acetylglucosaminyltransferase 7
*C19orf18*	−19,587	19	chromosome 19 open reading frame 18
*CALB1*	−39,948	8	calbindin 1, 28 kDa
*CALY*	−42,171	10	calcyon neuron specific vesicular protein (former *DRD1IP*)
*COL4A3*	−13,394	2	collagen, type IV, alpha 3 (Goodpasture antigen)
*CYP3A5*	−19,323	7	cytochrome P450, family 3, subfamily A, member 5
*DAPL1*	−19,323	2	death associated protein-like 1
*DNALI1*	−38,678	1	dynein, axonemal, light intermediate chain 1
*DNM1P46*	−20,693	15	dynamin 1 pseudogene 46
*DPCD*	−14,466	8	deleted in mouse model of primary ciliary dyskinesia
*DPF1*	−14,458	19	double PHD fingers 1
*EPOR*	−20,388	19	erythropoietin receptor
*FOXD4*	−14,149	9	forkhead box D4
*FZD9*	−18,458	7	frizzled class receptor 9
*KSR1*	−27,506	17	kinase suppressor of ras 1
*NIM1K*	−14,149	5	NIM1 serine/threonine-protein kinase
*PIGZ*	−25,072	3	phosphatidylinositol glycan anchor biosynthesis, class Z
*RAB11FIP1*	−13,097	8	RAB11 family interacting protein 1
*SLC9A9*	−22,712	3	solute carrier family member 9
*SSTR5*	−15,488	16	somatostatin receptor 5

**Table 5:** Features of female and male patients with Cushing’s disease.

Parameter	Female Patients (*n* = 122)	Male Patients (*n* = 31)
age (years)	38.9 ± 1.27	44.0 ± 2.89 *
microadenoma (% entire series)	56.3%	50%
invasiveness (% entire series)	9.8%	12.5%
surgical remission (% entire series)	69.2%	67.0%
recurrence (% remission series)	4.1%	3.2%
ACTH staining (% cells)	87.2 ± 1.36	87.1 ± 2.07

* *p* < 0.05 vs. specimens from female patients.
